# Translating evidence into practice: the role of health research funders

**DOI:** 10.1186/1748-5908-7-39

**Published:** 2012-04-24

**Authors:** Bev Holmes, Gayle Scarrow, Megan Schellenberg

**Affiliations:** 1 Michael Smith Foundation for Health Research, , 200-1285 West Broadway, Vancouver, BC, V6H 3X8, Canada

**Keywords:** Knowledge translation, Health research, Funding agencies, Evidence-informed practice, Evidence-informed policy

## Abstract

**Background:**

A growing body of work on knowledge translation (KT) reveals significant gaps between what is known to improve health, and what is done to improve health. The literature and practice also suggest that KT has the potential to narrow those gaps, leading to more evidence-informed healthcare. In response, Canadian health research funders and agencies have made KT a priority. This article describes how one funding agency determined its KT role and in the process developed a model that other agencies could use when considering KT programs.

**Discussion:**

While ‘excellence’ is an important criterion by which to evaluate and fund health research, it alone does not ensure relevance to societal health priorities. There is increased demand for return on investments in health research in the form of societal and health system benefits. Canadian health research funding agencies are responding to these demands by emphasizing relevance as a funding criterion and supporting researchers and research users to use the evidence generated.

Based on recommendations from the literature, an environmental scan, broad circulation of an iterative discussion paper, and an expert working group process, our agency developed a plan to maximize our role in KT. Key to the process was development of a model comprising five key functional areas that together create the conditions for effective KT: advancing KT science; building KT capacity; managing KT projects; funding KT activities; and advocating for KT. Observations made during the planning process of relevance to the KT enterprise are: the importance of delineating KT and communications, and information and knowledge; determining responsibility for KT; supporting implementation and evaluation; and promoting the message that both research and KT take time to realize results.

**Summary:**

Challenges exist in fulfilling expectations that research evidence results in beneficial impacts for society. However, health agencies are well placed to help maximize the use of evidence in health practice and policy. We propose five key functional areas of KT for health agencies, and encourage partnerships and discussion to advance the field.

## Background

A growing body of work on health-related knowledge translation (KT) reveals the significant gaps between what is known to improve health, and what is done to improve health. The costs of ignoring these gaps are increasingly apparent: suboptimal or unnecessary care, overuse or premature adoption of treatments, and new research that may not be based on the latest evidence or may not adequately address patient or other research users’ needs [1-5].

Fortunately, the literature on and practice of KT—defined by Canadian Institutes of Health Research (CIHR) as a dynamic and iterative process that includes synthesis, dissemination, exchange, and ethically-sound application of knowledge to improve the health of Canadians, provide more effective health services and products and strengthen the healthcare system [6]—also demonstrate its potential to narrow those gaps, leading to more evidence-informed healthcare.

The leadership of CIHR and Canadian Health Services Research Foundation (CHSRF), among others, has advanced the KT field considerably in Canada. Health organizations are making KT a priority, in part to demonstrate accountability for spending public dollars, but also recognizing they are ideally placed, due to their mandates—and also in the case of many health research funding agencies, their provincial reach—to help facilitate evidence-informed practice and policy making.

The purpose of this article is two-fold: first, to describe how one funding agency, the Michael Smith Foundation for Health Research (MSFHR) in British Columbia (BC), Canada, developed a plan to increase its role in KT; and second, to present a conceptual model we developed that health organizations could use when considering KT programs. The model comprises five key functional areas that together create an environment that best facilitates the use of evidence in healthcare practice and policy. It will likely not be appropriate or possible for agencies to engage in all of these areas. However, consideration of the extent to which and how they are being undertaken by others will increase the likelihood of developing successful KT initiatives.

The article will discuss the evolution of funding agencies before describing MSFHR and our KT planning. We then present our model of the five key functional areas of KT, provide examples of activities within each area, and discuss how we decided which areas to focus on initially. We end with considerations for health KT-focused organizations as they develop programs to help maximize the use of health research evidence.

## Discussion

### Health research funding agencies and the KT (r)evolution

In ‘Speeding up the Spread: Putting KT research into practice and developing an integrated KT collaborative research agenda,’ Kitson and Bisby [7] note how the 1998 Canadian federal budget documents justified an increase for health research: ‘To provide research grants, scholarships and fellowships for advanced research and graduate students.’ Ten years later, additional wording was, ‘The granting councils will partner with public and private stakeholders to ensure that practical solutions are found.’ The federal government was acting on a growing realization that funding excellent scientists to conduct their own programs of research is not enough; research must demonstrate a return on investment by addressing the health priorities of Canadians. Although ‘excellence’ determined through rigorous peer review is an important criterion by which to evaluate and fund research, it alone does not necessarily ensure the funding of research that is relevant to societal health priorities [8].

In 2009, The Canadian Academy of Health Sciences (CAHS) responded to increased government expectations by releasing an article entitled, ‘Making an Impact: a preferred framework and indicators to measure returns on investment in health research’ [9]. While many funding agencies use the framework to evaluate their programs, it is becoming clear that the returns they are looking for are not automatic. There is a need to move away from the traditional ‘fund and forget’ model [10] and review their funding priorities, grant review criteria, and research practices [11], and generally become more active in the space between research results and impact. As Kitson and Bisby suggest, ‘Greater involvement of funding agencies in all forms of KT is not just the right thing to do: it is essential for the maintenance of the health research enterprise in the face of many competing and compelling demands on the tax base.’ [7]

A review of Canadian provincial health research funding agency websites suggests they are responding enthusiastically to this challenge. KT activities include: province-wide initiatives targeted at researchers, policy makers, practitioners, and the public; peer-reviewed funding for researchers’ KT activities; KT science grants; KT education; KT networks; and KT conferences. A national initiative is also underway to build capacity in both the practice and science of KT [4].

Despite this increased focus, funders are still challenged by the sheer complexity of the KT enterprise. Tetroe *et al.*[12] explored these challenges in an international study that looked at: funding agencies’ expectations of their funded researchers; perceptions of their role in promoting the results of their funded research; activities toward promoting the use of the research they fund; and capacity to support KT. Agencies reported difficulty with: determining what to address in the KT agenda versus what other agencies should address; establishing a clear purpose; evaluating investments in KT; identifying reviewers who understand KT; getting recognition that KT takes time; and defining a systematic approach to their KT initiatives. These interviews were conducted in 2003 and 2004, but their findings are still relevant in our experience.

Kitson and Bisby [7] acknowledge these difficulties but suggest that funding agencies could do much more to support KT, including:

1. Require the involvement of research users throughout the research cycle;

2. Support activities to increase the ability of researchers to communicate with users;

3. Provide forums for knowledge users and researchers;

4. Require a KT plan for funded projects;

5. Provide training and support to review panel members for the assessment of a KT plan;

6. Include KT costs as eligible expenditures;

7. Fund activities that facilitate easier access to research data by knowledge users;

8. Require open access publishing;

9. Fund rapid response programs to address urgent health issues.

Furthermore, Tetroe *et al.*[12] suggest funders could clearly define what their definition of KT is so they are better able to develop their own KT strategy for funding implementation research; undertake KT themselves, including the dissemination of their funded research; and involve end users in prioritizing research topics for funding.

Considering the limited funding generally allocated to KT—described as ‘decimal dust’ by Kerner *et al.*[13]—funding agencies also need to expand their partnerships (non-government organizations, government, and private sector organizations) in order to leverage limited human and financial resources, and align with and build on existing and new KT initiatives and KT research. Kerner [2] stresses that leaders of research institutions and academic medical centres must ‘…reach out to the broad set of clinical services delivery leaders, all of whom compete for and receive resources from funding agencies, to develop new opportunities for research-practice partnerships.’ Collaborative development of priorities, he argues, may be the only way to ensure that an adequate investment is made in KT.

To determine how best to support the use of health research evidence in practice and policy within the existing complex KT enterprise in Canada, our agency initiated a planning exercise. Below we provide an overview of The Michael Smith Foundation For Health Research (MSFHR) and describe the exercise.

### The Michael Smith Foundation for Health Research

Named after Nobel Laureate Dr. Michael Smith, MSFHR was established by the BC government in 2001 with a $110 million dollar grant to strengthen the province’s health research enterprise. Our programs fund individuals (established researchers and those in training), research teams, and research projects that address healthcare and health system priorities.

Some KT elements are featured in most MSFHR programs. However, as demonstrated in Table 1, it was not always clear how or to what extent the KT elements were effective. The purpose of our KT plan was therefore two-fold: to improve KT activities related to our existing funding programs; and to work with partners to strengthen the province’s health KT enterprise overall.

**Table 1:** MSFHR Programs and Knowledge Translation (KT) Elements (as of March 2012)

**Program**	**KT element**	**Evaluation**
Health Services and Policy Research Support Network		
Health Authority Capacity Building Program: grant to facilitate participation in health services and policy research and evaluation activities	Overall KT focus	Health authority execs reported more evidence informed decision-making; staff reported that research evidence was used to improve services and programs
Investigative Teams Program: funding for five teams of researchers and decision makers		Team structure supported the conduct and uptake of research
Operating Grants Program: research to evaluate or inform health system redesign		Most findings disseminated; some were used to make decisions
BC Nursing Research Initiative		
Nursing Research Facilitator Program: funding for facilitators to act as researcher contact, help staff use evidence	Overall KT focus	Evaluations in development or underway
Nursing Health Services Network: brings together academic, practice and policy communities to advance nursing research		
Funding programs: research projects, investigative teams, partnership research, commissioned research		
Team Awards		
Research Unit Awards	Requirements include collaborative research activities to address health system priorities; dissemination	Until recently, annual reports from the units requested only basic information on KT activities
Research Team Start-Up Awards		
Research Team Planning Awards		
Networking Awards		
Health of Population Networks: eight networks of health researchers with a common interest in specific populations	KT focus	Evaluation framework not developed until halfway through awards but collectively the networks developed a knowledge exchange plan, report [14] indicated they increased the quality, quantity and impact of health research in BC.
Technology/Methodology Platform Awards: helped establish five provincial cross cutting platforms that support a range of health research applications	Some KT requirements	A range of KT activities reported, including guideline and best practice development, training, public engagement, and online resources
Personnel Programs		
Awards to support researchers from trainees to established investigators	Limited KT	Information on KT activities requested only recently in annual reports, which gather data on end user engagement and dissemination

While much of KT focuses on the use of evidence in policy and practice settings, equally important for MSFHR is maximizing the impact of basic and clinical research, which may have less immediate relevance from a healthcare or health systems perspective, but the findings from which could inform subsequent research in various fields. As a research funding agency, our focus is evidence from health research studies. However, this narrow perspective of ‘evidence’ [15] was our starting point only; we emphasize in our planning assumptions (Table 2) the complexity involved in the implementation or use of study findings, including the different types of evidence and conceptualizations of knowledge use [16] that must be brought to bear.

**Table 2:** Knowledge Translation (KT) planning assumptions

**KT planning assumptions**	
**1** KT for MSFHR focuses on research-generated evidence with the intent of maximizing its use, whether applying it to further research, policies, products, practices, or even making a decision *not* to undertake one of those actions based on the findings.	
**2** KT involves interactive, non-linear, social processes underpinned by effective exchanges (of evidence, ideas, expertise, information and opinions) among creators and users of research evidence.	
**3** Research users include the general public, patients, other researchers, health professionals and administrators, policy-makers and industry.	
**4** KT is an increasingly important practice within the research process *and* a scientific discipline.	
**5** Rather than consisting of a unified theory and practice base, KT draws on a range of theories and practices depending on the project underway.	
**6** KT focuses on ‘knowledge’ (which requires internalization and understanding) as opposed to ‘information’ (organized data).	
**7** Improving the availability of evidence does not ensure its use: a range of goal-oriented, audience-specific KT strategies is needed to maximize the impact of health research.	
**8** KT often requires a focus on practice-based implementation research, which explores the scaling up of interventions in ‘real world’ contexts.	
**9** At times, KT requires a systems thinking approach to connect analytical problem solving with the chaotic and complex ways in which change takes place in social practice.	
**10** The context in which KT occurs must be considered: not only existing knowledge (tacit and explicit), beliefs, attitudes, values and opinions of researchers and users, but also institutional arrangements and culture, political interests, resources, geography, power and influence over decision-making.	
**11** Evaluating the effectiveness of KT is a methodological challenge but critical.	
**12** Health research funding agencies can play an important role in KT.	

### MSFHR knowledge translation planning

We began our KT planning exercise by: developing assumptions (Table 2) before conducting an environmental scan including assessing our current KT activities (Table 1); interviewing stakeholders involved in KT across Canada and in BC health authorities, not-for-profit agencies, government and the research community; reviewing KT literature; and exploring websites of health-related KT organizations. Interviewees were asked about how they conceptualized KT, details of KT activities they were doing, what gaps they saw in the field, who else we should speak with, and what KT-related websites we could explore. We then searched these websites and collected information on the programs and resources they described.

Based on initial findings, we wrote and circulated the first version of a discussion paper to support an inclusive and iterative process for gathering feedback from stakeholders consulted as part of the initial environmental scan. The paper presented opportunities for MSFHR’s overall role in KT based on the roles of other organizations. An approach to framing our KT plan was proposed and stakeholders were encouraged to suggest next steps towards developing the plan as well as providing considerations for focusing the next stages of work. Feedback was incorporated and a version sent back to interviewees and the additional people they had identified asking for additional feedback on the most debated topics. These included:

1. What is the importance of KT to the biomedical and clinical research ends of the research spectrum?

2. What is the overall purpose of KT—to maximize impact? Relevance? Use? All three?

3. Should MSFHR work towards a provincial KT plan in partnership with the provincial government?

4. Is the public a focus for funders’ KT activities?

5. How feasible is it to measure or expect funders and/or grant holders to monitor and evaluate research impact that takes expertise, time, and cost?

6. Is there a role for funders to facilitate a culture shift among researchers, decision makers, universities, allied health professionals, industry, and the public so that KT is rewarded appropriately and recognized for the time it takes?

7. How is KT best funded when thinking in broad terms of maximizing relevance, use and impact of health research?

The paper continued to be circulated among approximately 60 stakeholders until no new comments were forthcoming. The final version of the discussion paper can be found on our website [17].

Key to the discussion paper were five key functional areas we identified in which funding agencies can work. A KT expert working group, convened to review the discussion paper, its five key functional areas and a draft planning framework, was asked to provide recommendations for an overall KT approach for MSFHR, as well as activities for years one, two and beyond. Pre-meeting documentation sent to participants included MSFHR’s goals and the BC Ministry of Health’s innovation and change strategy. The group’s deliberations resulted in advice on where best and how to move forward on the development of a draft organizational KT plan in four broad areas that were deemed both the biggest gaps and the easiest to experience some short terms success with and therefore build momentum: knowledge to action demonstration projects; capacity building (skills and knowledge needed for KT); partnerships (especially related to co-funding and co-implementation with organizations that have similar areas of interest, which in our experience not only leverages funds but increases commitment and therefore likelihood of health evidence use); and enhancing KT in new MSFHR programs and developing a tracking system for existing programs (for example, to highlight KT best practices in order to share them, to surface and potentially support promising research that is ready to be taken to the next step, and to connect researchers and teams across funding programs).

Finally, in order to better understand currently available KT training and resources for our capacity-building priority, we contracted a research group to gather additional information on KT education and training and KT funding programs that could be used as models as well as be assessed overall for partnership opportunities and for gaps. Based on the expert working group’s recommendations and this additional information and aligned with our immediately available resources, we finalized a plan, the first year of which focused primarily on the functional area of building KT capacity, with some emphasis on funding and advancing KT science.

The goals and first year objectives of our KT plan are outlined in Table 3. Tables 4 and 5 list our critical success factors and examples of program criteria, respectively. The critical success factors were developed to guide the roll-out of the plan, and the criteria to help us determine which activities to undertake, given the seemingly unlimited need for KT supports, the great number of opportunities, and our limited resources. The criteria can be adapted slightly depending on the type of activity under consideration.

**Table 3:** MSFHR KT goals and first year objectives

**KT Goals 2011 to 2015**	
1. Build KT skills of BC researchers and research users	
2. Bring synthesized evidence to bear on resolving BC health system issues	
3. Maximize the impact of MSFHR-funded research	
**First Year Objectives 2011 to 2012**	
1. Sponsor two researcher workshops	
2. Sponsor two research user workshops	
3. Fund two KT practice to science demonstration projects	
4. Strengthen KT requirements of MSFHR funding programs	
5. Develop and implement internal KT support structures and processes	
6. Conduct a provincial KT needs assessment	

**Table 4:** Critical success factors for MSFHR’s KT plan

• We define ‘knowledge translation’ consistently, using plain language.	
• The BC health research community understands the importance of KT, and the value of their engaging in it outweighs the cost.	
• We are seen to be credible and influential as a KT organization in BC and across Canada.	
• Our KT function is adequately resourced, and we leverage our KT budget.	
• Our KT activities are specific in their audiences and objectives such that they can be rigorously evaluated; they are based on good evidence themselves, we learn from our KT activities, and disseminate lessons learned.	

**Table 5:** Examples of criteria for KT activities

**The activity will:**	
• Address a demonstrated need	
• Not duplicate existing programs	
• Maximize our resources through co-funding from a partner	
• Increase our KT profile or that of our funded-researchers	
• Strengthen the KT community	
• Address provincial health/health system priorities	

### KT key functional areas and observations

In the process of developing our plan, we identified the following five key functional areas that together create an environment that facilitates effective use of evidence in practice and policy making. These five areas were determined as categories to encompass the range of KT activities offered by funders. One agency probably cannot work in all these functional areas in a comprehensive way, whether due to mandate or resource constraints, but ideally they would all be considered as part of the planning context. As well as describing the key functional areas below, we present a number of observations for consideration in a KT planning exercise.

### Five KT key functional areas for funding agencies

**Figure 1:**
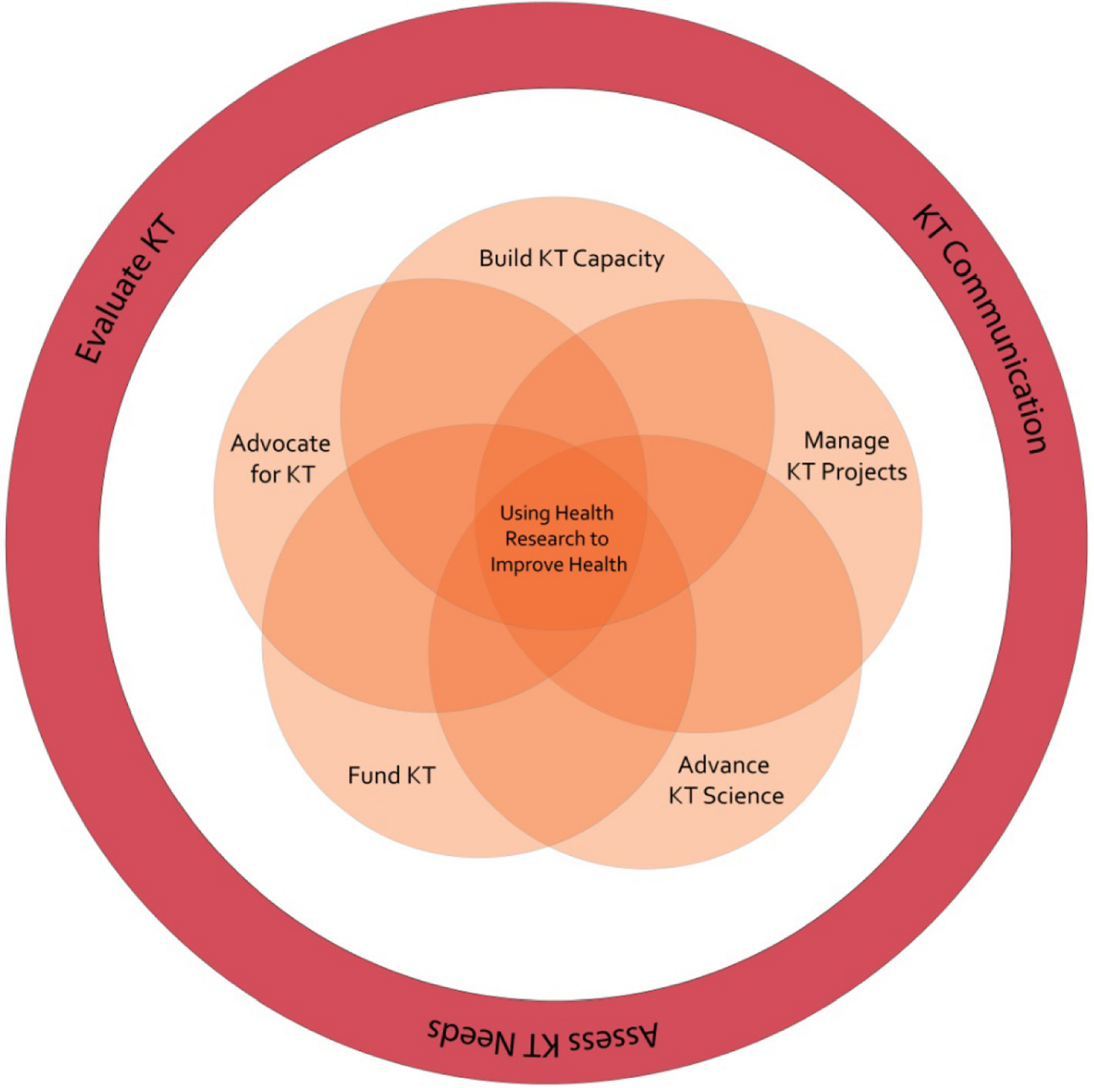
Key functional areas for agencies involved in KT.

KT activities from a funder’s perspective fall into five key functional areas: advancing KT science, building KT capacity, managing KT projects, funding KT activities, and advocating for KT [Figure 1]. The areas are not necessarily discrete (*e.g.*, a funder could both fund and manage a KT activity), but the primary objective usually falls under one functional area. As Figure 1 shows, additional activities to support a KT program are: assessment of stakeholder KT needs to identify gaps and opportunities and avoid duplication of efforts; evaluation of KT activities (the funders’ as well as their funded researchers) for outcomes and impacts and to provide opportunities for course corrections and to collect lessons learned; and communication of KT activities (funders’ and their funded researchers). The key functional areas and examples of KT activities for each are outlined below.

### Advancing the science of KT through research

Advancing the science of KT through research is, according to stakeholders consulted and the literature, one of the biggest gaps in KT [18,19]. While a number of theories, methods and mechanisms have been proposed for KT activities, there is a lack of research evidence to determine the best context for their use. For this reason, advancing the science of KT is an important focus for a health research funding agency. Examples of funding agencies’ activities in this area include: evaluating, monitoring, and/or publicizing the impact of evidence use; adding to the KT literature by developing papers, presentations or casebooks; participating in development and implementation of provincial and national KT science projects; funding KT science grants and projects; and hosting KT conferences.

### Building capacity for KT

Building capacity for KT is defined by MSFHR as developing skills and providing tools to maximize the impact of health research. This functional area involves active planning, management and evaluation of KT capacity building activities by the funder either alone or with partners. Examples of funding agencies’ capacity building activities include:

1. Audience-specific training (*e.g.*, workshops) on KT overall as well as aspects of KT, such as knowledge syntheses, interpretation of evidence, evaluating KT efforts, working with decision-makers, working with researchers;

2. Access to KT resources: help-desk; hosting a provincial KT network; providing or linking to resources such as KT models, tools and best practice information; providing links to review databases (*e.g.*, healthevidence.ca, healthsystemevidence.ca, Cochrane Collaboration);

3. KT accreditation programs for health professionals and KT professionals.

### Management of KT projects

Funders have traditionally not been involved in the ‘doing’ of KT themselves, but are increasingly becoming involved in such activities. We identified an opportunity to become more hands-on with some of our funding programs, helping researchers and decision-makers to set KT goals and participating in their KT activities as appropriate, or assisting with evaluating the outcomes. Funders are also:

1. Organizing forums where researchers and research users discuss a specific issue (clinical/service/policy) with a view to developing and implementing a solution;

2. Managing KT projects from start to finish (knowledge generation to implementation and monitoring, (*e.g.*, clinical guidelines or healthcare policies);

3. Managing one or more steps in the knowledge-to-action cycle: knowledge synthesis, implementation (*e.g.*, of existing guidelines), targeted dissemination and evaluation of products (*e.g.*, discussion papers).

### Funding KT

Where the funder provides the financial support to individual researchers or teams of researchers (teams may include research users) for their KT activities. Examples from funding agencies include:

1. Funding knowledge brokers (liaison between researchers and users) and/or research on the role and effectiveness of knowledge brokering;

2. KT supplement grants for existing awards;

3. Funding for KT model testing or other KT research project grants;

4. Providing awards for research use and uptake (*e.g.*, to adapt and implement research evidence);

5. Funding for knowledge syntheses;

6. Funding awards for ‘rapid response’ solutions, which would allow expert research teams to provide timely solutions to urgent health issues identified by research users.

### Advocating for KT

Where the funder actively addresses barriers to effective KT and influences change through such activities as:

1. Presentations, publications, position papers, calls to action on specific health/health system issues;

2. Influencing/enabling KT (*e.g.*, by celebrating the work of researchers and others who are promoting a climate that fosters KT; encouraging culture change within colleges and universities to recognize and credit KT accomplishments in faculty promotion and tenure deliberations);

3. Mandating KT as part of funders’ programs and activities, as appropriate (*e.g.*, required as part of awards — in many cases, this involves training applicants and reviewers on KT requirements);

4. Developing a culture of KT internally.

In terms of our own KT plan, the areas and activities identified for year one were chosen to address two groups of stakeholders in BC: those with experience in incorporating KT into their research and/or work (addressing the key functional areas of ‘funding KT’ and ‘advancing the KT science’), and those with some or limited understanding of KT (addressing the key functional area of ‘building KT capacity’). We felt that we could achieve ‘quick wins’ in these areas with our current resources while developing the internal and external resources and partnerships needed towards the development and implementation of year two KT objectives. A KT needs assessment survey related to KT skills training and resources that targets provincial researchers and those who use health research evidence in their work was launched (March 2012) to inform year two plan development.

## Observations

During our KT plan development, we made the following observations of relevance to the KT enterprise as a whole. We describe them here briefly for the consideration of agencies developing KT plans.

### The difference between KT and communications

Many health research websites, publications and initiatives conflate communications and KT despite the differences between those endeavors. Communications is an important aspect of KT—and an important practice distinct from KT—but KT goes beyond communications. Many of the so-called KT activities we found during our environmental scan were actually communications activities, for example, media and publicity, health communication awards, and information dissemination through websites or materials. While such activities are critical supports for KT, in isolation they do not ensure the translation of knowledge into practice. Boaz *et al.*[20] conducted a review of systematic reviews on the effectiveness of interventions in clinical practice designed to increase the use of research. The reviews suggest that active, multi-faceted interventions (*e.g.*, feedback, opinion leaders, and reminders) are more effective than passive approaches (*e.g.*, information campaigns). Similarly, Fixsen *et al.*[21] concluded, based on an analysis of 377 publications, that information dissemination alone is not effective as far as implementation.

Lervik *et al.*[22] note that a ‘syntactic’ approach, which conceives of knowledge transfer as the process of sending and receiving messages (in other words, a communications approach), may work in situations of highly shared context, for example, a specific research community. However in most cases, the implementation of research evidence into a new practice or policy necessitates the crossing of disciplinary and other types of boundaries, and requires the understanding of different perspectives, joint problem solving—with a recognition that not everyone perceives the same problem, and indeed that solving a problem in one area may create problems in another—and the co-development of new knowledge. Communications expertise can be helpful in these processes, but it is only one of many types of expertise needed.

To maximize their KT efforts, funding agencies need to distinguish between organizational communications activities *per se*, and communications activities as part of KT efforts. The latter should be an integral part of a purposeful, impact-oriented approach to maximizing the impact of health research.

### The difference between information and knowledge

There is a related tendency to conflate information and knowledge, which are different from each other. In our environmental scans and interviews, we frequently read and heard people talk about ‘disseminating knowledge,’ which we think should more appropriately be referred to as disseminating information, which in turn must become ‘known’ by a recipient—integrated into his or her existing understandings—to become knowledge [23]. It is increasingly recognized that developing technologies and systems to codify and share explicit knowledge (which relates closely to information) is not enough, and attention must be paid to the sharing of tacit knowledge [22] and to incorporating distinct forms of knowledge from multiple sources [16,24].

The idea of knowledge as distinct from data and information supports a much broader definition of KT than dissemination of research results [25] and has implications for funding agencies’ KT programs, which ideally would include activities such as facilitation of researcher-research user interactions and KT skills training for researchers and research users.

### Who is responsible for KT?

Another important question for funding agencies that rose from stakeholder feedback to our discussion paper is, ‘who is responsible for KT?’ Funding agencies can do much to support researchers and users in their KT efforts, including training on various aspects of KT, supporting them through KT expertise, or by providing funding at the appropriate time to secure KT expertise. To this end, we do not agree that all researchers—regardless of the type of research they conduct or the stage it is at—should be solely responsible for KT related to their work. While certain KT requirements should be expected of researchers, and ideally they would understand the importance of KT, it is not reasonable to expect them to develop the broad range of competencies and skills required to undertake thorough KT. Government agencies (*e.g.*, Public Health Agency of Canada, Health Canada, Indian and Northern Affairs Canada), health authorities, and research institutes—whose mandates often include generating and transferring scientific information—also have a role to play in supporting KT, and are increasingly dedicating resources to this role individually and collectively. Through inter-governmental, cross-organizational, and multi-jurisdictional collaborations—in a model of shared responsibility for KT—we are beginning to see a systemic change that should have a positive impact on program and policy development.

### Implementation and evaluation gaps

As noted in the literature [1-5] and underscored in our interviews, gaps exist in implementation capacity: even when the evidence is clear, it does not necessarily get used. Barriers exist at a number of levels, including individual (lack of awareness or familiarity with the evidence, lack of time, lack of skills) and organizational (lack of understanding of KT needs, political constraints, evidence that may not support current programs in which resources have recently been invested). Boaz *et al.*[20] found that very few systematic reviews look ‘exclusively and explicitly at implementing research findings into practice.’ Limited investment in implementation research—which includes evaluation of the implementation—leaves us with a lack of understanding of how to move evidence into practice and policy. Ideally, the evidence from the research study itself is integrated and synthesized with many other types of evidence: tacit and explicit, quantitative and qualitative, process and outcome, intended and unintended outcome data, adoption and sustainability, and more [15]. Certainly there are inherent challenges in evaluating the use of evidence: metrics are difficult to define and reach agreement on, the use of research evidence differs depending upon the context, it takes time and money to monitor and evaluate the impact of research use—and, in acknowledging that no single source of knowledge can provide definitive answers, we must also expect findings and insights to sometimes be contradictory [24]. Despite the challenges, funders working in partnership with individuals and organizations in both the research and research user communities are in a position to move things forward by: advocating for change in support of implementation of research evidence; providing KT skills training and mentoring or coaching for researchers and research users; providing targeted funding for KT implementation science projects; and managing implementation and evaluation initiatives.

### The rush to practice/policy

A final observation, or perhaps caution, is that not all research moves directly into policy or practice, and even when it does, it takes time. Under pressure to demonstrate results quickly, funders and researchers themselves often forget to underscore the important messages about the lengthy steps involved in the research cycle; the fact that research results should not be implemented in isolation of a larger body of knowledge; the cultural and political shifts that often need to take place in order for evidence to be used in a clinical, health practice- or policy-related decision; the wide range of evidence types that need to be integrated and the new forms of knowledge that need to be generated for successful implementation to take place, and the time needed to evaluate the change. With no ‘best timeframe’ for either conducting research or implementing its findings, funders need to be clear as to what realistic KT activities and/or KT impacts are expected from funded researchers—these will differ between funding programs even within the same organization. Funding schemes that are relatively short and do not take into account the complexities outlined above will fail to meet the funders’—and therefore stakeholders’—expected return on investment. Funders need to invest in moving knowledge into action so that it happens as quickly and efficiently as possible but they must also make clear that the processes of research itself (particularly when integrating stakeholder involvement into the research cycle) and the implementation of its findings is highly complex, and time-consuming. This is where the advocacy role can be particularly important for funding agencies.

## Summary

There are many challenges to fulfilling the increasing political and societal expectations that the results of research must lead to beneficial impacts for citizens. However, these are exciting times for health research funders and other organizations involved in KT as it becomes clear they are in an opportune position to meet these challenges by supporting the use of research evidence in health practice and policy making. For funding agencies, we believe the best way to do this is to develop a KT plan that views KT as a ‘complex system of interactions between researchers and knowledge users’ that varies depending upon the type of research, research findings and the needs of the user [26]. In other words, although a focus on evidence from health research studies is justified, putting the evidence to use demands a much broader view of what ultimately constitutes evidence for a range of stakeholder groups.

In our experience, the five key functional areas provided the ‘bigger picture’ of the KT enterprise, and helped us determine where best to expend our efforts during a first year of focused KT activities. We suggest that other health-related agencies interested in developing or expanding their KT efforts could take a similar approach to planning, using the model we developed as a framework. As a start, they could:

1. Draft a KT positioning statement describing how KT fits into their mandate, how they conceptualize it and any planning assumptions and parameters;

2. Conduct an internal assessment of KT activities in each of the five key areas—what are the activities and how successful are they?;

3. Conduct an external environmental scan on KT activities in each of the five areas;

4. Assess gaps within each area and across all areas.

The above information could be used to create an internal/external environmental scan report as a basis for developing a plan. There are any number of approaches that could be used to develop a plan, but based on our experience we suggest they include an expert working group and wide consultation. Ultimately ideal would be the collaboration of the various agencies involved in KT in a jurisdiction such that ‘who is best positioned to do what’ with regard to KT funding, science, advocacy, capacity development, and management could be negotiated and agreed on, and objectives and action plans could be developed collectively.

Funding agencies and health organizations involved in KT have much to learn from each other, and the healthcare system and its users have much to gain from a collective effort to maximize the use of health research evidence in practice and policy. Therefore, we welcome feedback on our ideas and encourage discussion and partnership among health agencies to advance the field of health KT.

## Competing interests

The authors declare that they have no competing interests.

## Authors’ contributions

BH co-wrote the paper. GS co-wrote the paper. MS provided input and revisions. All authors read and approved the final manuscript
